# Effectiveness of betadine-coating gastrostomy tube to reduce peristomal infection after percutaneous endoscopic gastrostomy: a randomized controlled trial

**DOI:** 10.1186/s12876-023-02702-w

**Published:** 2023-05-15

**Authors:** Yu-Jen Chen, Ming-Chih Hou, Tsung-Chieh Yang, Pei-Chang Lee, Yen-Po Wang, Yi-Hsiang Huang, Fa-Yauh Lee

**Affiliations:** 1 Division of Gastroenterology and Hepatology, Department of Medicine, Taipei Veterans General Hospital, No. 201, Sec. 2, Shih-Pai Rd., Taipei 112, Taipei, Taiwan; 2 National Yang-Ming Chiao-Tung University School of Medicine, Taipei, Taiwan, ROC; 3 Division of Endoscopy Center For Diagnosis and Treatment, Taipei Veterans General Hospital, Taipei, Taiwan

**Keywords:** Percutaneous endoscopic gastrostomy (PEG), Peristomal wound infection, Povidone-iodine

## Abstract

**Background:**

Peristomal wound infection is a common complication in patients receiving percutaneous endoscopic gastrostomy (PEG). The main reason for peristomal infection might be the oral microbes coating the gastrostomy tube during implantation. Povidone-iodine solution can be applied for skin and oral decontamination. We designed a randomized controlled trial to test the effectiveness of a Betadine® (povidone-iodine) coated gastrostomy tube to reduce peristomal infection after percutaneous endoscopic gastrostomy.

**Methods:**

A total of 50 patients were randomized to Betadine and control groups (25 patients in each group) from April 2014 to August 2021 at a tertiary medical center. All patients received the pull method for PEG implantation using a 24-french gastrostomy tube. The primary endpoint was peristomal wound infection rate 2 weeks after the procedure.

**Results:**

Changes in Neutrophil/Lymphocyte ratio (N/L ratio) and C-Reative protein (Delta CRP) at 24 h after PEG were higher in the control group than in the Betadine group (N/L ratio, 3.1 vs. 1.2, p = 0.047; CRP, 2.68 vs.1.16, p = 0.009). The two groups did not differ in post-PEG fever, peristomal infection, pneumonia, or all-cause infection. Delta CRP could predict peristomal infection and all-cause infection within 2 weeks (AUROC 0.712 vs. 0.748; *p* = 0.039 vs. 0.008). The best cut-off-point of Delta CRP for the diagnosis of peristomal wound infection was 3 mg/dl.

**Conclusion:**

The betadine coating gastrostomy tube method could not reduce peristomal infection after percutaneous endoscopic gastrostomy. CRP elevation of less than 3 mg/dl may be used to exclude the potential peristomal wound infection.

**Trial registration:**

NCT04249570 (https://clinicaltrials.gov/ct2/show/NCT04249570).

**Supplementary Information:**

The online version contains supplementary material available at 10.1186/s12876-023-02702-w.

## Introduction

Percutaneous endoscopic gastrostomy (PEG) was introduced by a pediatric surgeon in 1980 [1] to replace traditional surgical gastrostomy. It has been used widely in patients with dysphagia and the need for enteral feeding. There are three types of PEG implantation methods: pull method, push method, and introducer method. Of these, the pull method is the most common [2, 3].

Compared to traditional surgical gastrostomy, there were fewer complications and a lower mortality rate in patients receiving PEG [4, 5]. However, there were significant complications with PEG. One of the most common complications of PEG was peristomal infection [6, 7]. The main reason for peristomal infection might be the oral microbes coating the gastrostomy tube during implantation [8]. Although the Cochrane review in 2013 revealed that prophylactic antibiotics could reduce the risk of peristomal infection in patients undergoing PEG placement, there was still a considerable number of peristomal infections, about 5.2–32.1% [9]. A prospective randomized controlled trial indicated that using an over tube might reduce the peristomal infection rate [10]. This method was not routinely used to consider the possible complication of over tube insertion such as esophageal rupture, mucosal laceration, or submandibular abscess.

Povidone-iodine is a chemical complex of povidone, hydrogen iodide, and elemental iodine. It had been applied in hand disinfection, skin preparation, and antiseptic irrigation [11]. Povidone-iodine is a relatively safe chemical complex. Common side effects of povidone-iodine include local swelling, itching, or rash. However, some case reports showed that it might be related to thyroid dysfunction and kidney injury in high dose retention [12, 13]. Several retrospective studies have indicated that oral irrigation with povidone-iodine could reduce infection rates in patients receiving dental extraction procedures or gingivectomy [14, 15], but no prospective study was conducted.

It is rational to expect that povidone-iodine coating gastrostomy tube would help reduce opportunities for oral microbes’ colonization and reduce the peristomal infection rate. We designed a randomized controlled trial to test the effectiveness of a povidone-iodine coating gastrostomy tube to reduce peristomal infection after percutaneous endoscopic gastrostomy.

## Method

### Patients

This randomized controlled trial was designed to test whether Betadine (10% povidone-iodine) coating on the PEG tube before implantation could reduce the peristomal infection rate. All patients were referred to the endoscopy unit for PEG implantation from April 2014 to August 2021 at a tertiary medical center. The study had been approved by the Institutional Review Board of Taipei Veterans General Hospital (VGHIRB No. 2013-11-017 C) and trial registration identifier: NCT04249570, ClinicalTrial.gov, 30/01/2020.

Patients were excluded from the study if they met the following criteria: the patient’s age was younger than 18 years or older than 100 years; the patient was not eligible for PEG implantation with pull methods, such as massive ascites, coagulopathy, malignancy with stomach invasion, severe left side hepatomegaly, high transverse colon, esophageal stenosis, thyroid dysfunction, history of upper abdominal surgery, or intra-abdomen bleeding; allergy history to cephalosporin or povidone-iodine; antibiotics use within 48 h due to infection, and the patient had an active infection. Randomization assignments were computer-generated and not announced until the trial was completed. To ensure blinding, treatment assignments were contained in sequentially numbered opaque sealed envelopes, which were opened by an independent research staff immediately after the patients’ eligibility was confirmed.

### PEG procedure

All patients fasted for at least 6 h before PEG implantation. Intravenous cefazolin (1000 mg infusion) and analgesics with meperidine HCl (50 mg/ml) 0.8ml or tramadol HCl (100 mg/2 ml) 2 ml were given 30 min before the procedure. Oral cleaning with a sterile cotton swab by the nurse would be administered before the procedure.

All patients received the pull method for PEG implantation with a 24-french gastrostomy tube (Percutaneous Endoscopic Gastrostomy Systems, Wilson Cook, Bloomington, Indiana, USA). The gastrostomy tube in the experimental group would be coated with Betadine (povidone solution 10%, Taiwan Veterans Pharmaceutical CO., LTD. Taiwan) via soaked sterile gauze before implantation, and the tube in the control group was coated with saline-soaked gauze only.

We performed esophagogastroduodenoscopy before the PEG procedure. The stomach would be inflated by air to ensure the stomach’s anterior wall could attach to the abdominal wall. Then, we turned on the light and checked the transmission light source at the skin site on the belly. We used fingers to push the belly and tried to find the optimal stoma site. After the stoma site is decided, we use Betadine and 75% alcohol for skin disinfection. After local analgesics with xylocaine 5 ml, a stoma was created by sterile knife and trocar. The guided wire was sent in the trocar and grabbed by a snare. The snare was tied with a PEG tube, and the tube was pulled down through the mouth, into the stomach and out through the abdominal wall, then anchored with an external bolster. We also recorded the PEG procedure times.

### Stomal wound evaluation (Fig. 1)

**Fig. 1:**
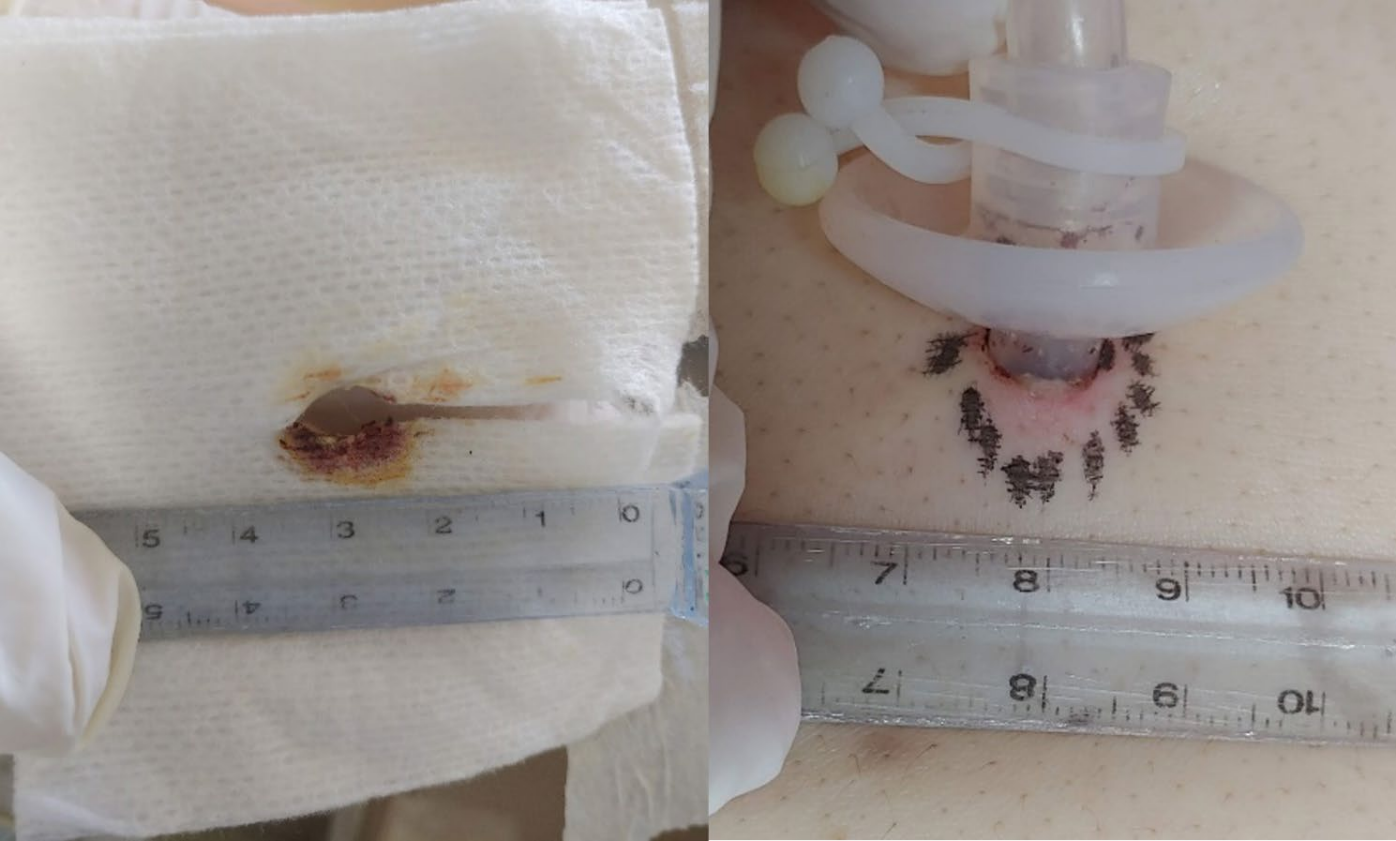
Peristomal wound infection evaluation Erythema about 2 mm, score = 1; induration score = 0, serous exudate, score = 1, total wound score = 2

After the PEG procedure, the patient received wound dressing with dry gauze and breathable adhesive dressing every day. The stomal wound would be evaluated by one gastroenterologist, unaware of the trial group, every day for 1 week and on days 10 and 14. Peristomal wound infection was evaluated by photos (under flashlight) with score system developed by Jain et al. [16], which include erythema (0 = none, 1 = < 5 mm, 2 = 6 to 10 mm, 3 = 11 to 15 mm, 4 = > 15 mm), induration (0 = none, 1 = < 10 mm, 2 = 11 to 20 mm, 3 = > 20 mm), and exudate (0 = none, 1 = serous, 2 = serosanguinous, 3 = sanguineous, 4 = purulent). For the patients discharged within 2 weeks after PEG implantation, their families were asked to change dressing and photograph wound conditions and send them to our study group. Diagnosis of peristomal infection was established if a total score ≧ of 8 or the presence of purulent discharge. Stomal leakage was defined as milk leakage on gauze within 2 weeks after PEG insertion. Oral cavity microbes’ culture was obtained before the procedure; wound culture would be obtained if the peristomal infection was impressed. Blood tests, including complete blood count, renal function, hepatic function, blood culture and CRP, were checked the day before and the next day of the PEG procedure. Post PEG fever was defined as body temperature over 38℃ within 2 weeks after the procedure. The diagnosis of pneumonia would be established if there was newly found infiltration on chest X-ray and infectious signs, such as leukocytosis, fever, or elevation of serum CRP level within 2 weeks after PEG implantation. Changes in N/L ratio and CRP (Delta CRP) were recorded the next day after PEG. Admission days were recorded from the PEG implantation date to the discharge date.

### Statistical analysis

The primary endpoint was peristomal wound infection rate 2 weeks after the procedure. The secondary endpoint includes wound positive culture rate, blood culture positive rate, pneumonia, variation of white blood cell, and CRP, PEG, procedure time and stomal leakage rate. The Fisher exact test or a χ2-test with Yates’ correction was used to compare categorical variables when appropriate, and the Mann-Whitney U-test was used to compare continuous variables. The risk factors of peristomal wound infection were compared using the Logistic regression model.

The variables with statistical significance (P < 0.05) or approximate significance (P < 0.1) by univariate analysis were subjected to a multivariate analysis using a forward stepwise logistic regression model. A two-tailed p-value of less than 0.05 was considered statistically significant. The diagnostic accuracy of delta CRP was examined by the receiver operating characteristic curve (ROC curve). The best cut-off value of each indicator was chosen based on Youden’s index. The sensitivity, specificity, negative predictive value (NPV), and positive predictive value (PPV) were calculated based on the cut-off point. All statistical analyses were carried out using IBM SPSS Statistics for Windows, version 23.0 (IBM Corp., Armonk, New York, USA). Peristomal infection within 2 weeks after PEG implantation was the outcome measurement to estimate the sample size for this study. Assuming an alpha risk of 0.05, a beta risk of 0.8, and peristomal infection to be 5% in the Betadine group and 20% in the control group according to previous studies [9, 10], the number of patients needed in each treatment group was estimated to be 50. Interim analysis was performed after half of the estimated cases were reached. Futility testing revealed it was unlikely to achieve statistical significance when 50 patients were enrolled, so we decided to terminate the study.

## Results

### Clinical characteristics of patients undergoing PEG

**Fig. 2:**
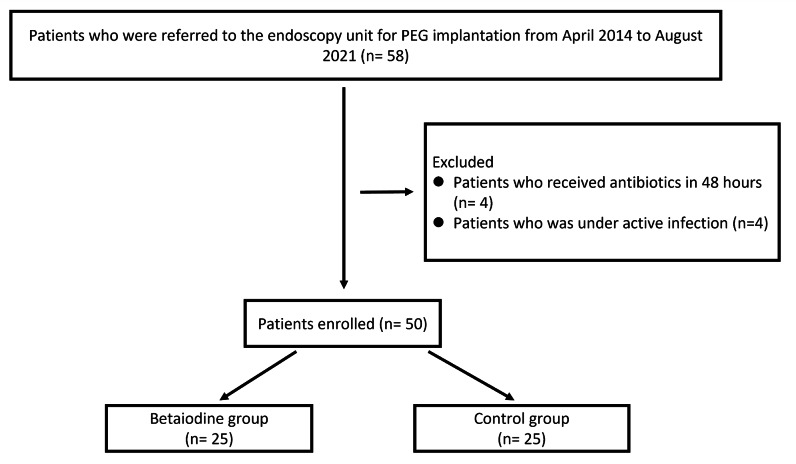
Patients’ flow of inclusion

From April 2014 to August 2021, a total of 58 patients were assessed for eligibility; eight were excluded (Fig. 2), and 50 were randomly assigned to receive either PEG with Betadine coating (n = 25) or not (n = 25). One patient in the control group expired on day 6 after PEG implantation due to pneumonia. There was no significant difference in patients’ baseline characteristics between the Betadine and the control groups. There was a trend that serum levels of WBC, CRP and N/L ratio were higher, with shorter PEG procedure time in the control group. Changes of N/L ratio and CRP (Delta CRP) were significantly higher in control group than Betadine group (N/L ratio, 3.1 vs. 1.2, p = 0.047; CRP, 2.68 vs.1.16, p = 0.009) (Table 1). The two groups did not differ in post-PEG fever, peristomal infection, pneumonia, or all-cause infection.

**Table 1:** Demographic data

Patient Demographic	All (N = 50)	Betadine (N = 25)	Control (N = 25)	p value
Age (years)	69.5 (55.7–86)	73 (55-87.5)	69 (55.5–83)	0.712
Sex (M/F) (%)	29 (58%)/21 (42%)	14 (58.3%)/10 (41.7%)	15 (57.7%)/11 (42.3%)	0.963
BMI	19.99 (17.89–23.23)	18.98 (17.38–23.06)	21.30 (18.41–23.82)	0.443
Head and neck cancer (%)	7 (14%)	5 (20%)	2 (8%)	0.417
CVA (%)	17 (34%)	6 (24%)	11 (44%)	0.232
HTN (%)	28 (56%)	14 (56%)	14 (56%)	1.000
DM (%)	14 (28%)	6 (24%)	8 (30.8%)	0.754
Dementia (%)	17 (34%)	9 (36%)	8 (32%)	0.765
ALS (%)	15 (30%)	6 (24%)	9 (36%)	0.355
Biochemistry				
Albumin-before (g/dl)	3.7 (3.3–4.1)	3.7 (3.0–4.0)	3.7 (3.3–4.1)	0.690
WBC-before (cumm)	6.8 (5.8–8.4)	6.3 (5.4–8.3)	7.5 (6.1–8.5)	0.252
N/L ratio-before	3.2 (2.6–4.4)	3.2 (2.2–4.3)	3.3 (2.6–4.6)	0.778
CRP-before (mg/dl)	0.27 (0.09–1.07)	0.27 (0.09–1.15)	0.23 (0.09–1.24)	0.854
WBC-after (cumm)	9.0 (6.8–10.6)	8.3 (6.2–10.2)	9.3 (8.1–11.5)	0.099
N/L ratio-after	5.5 (3.5–7.4)	4.6 (3.1–6.4)	6.3 (3.6–8.1)	0.052
CRP-after (mg/dl)	2.64 (1.06–5.01)	1.91 (0.79–3.14)	3.62 (1.52–5.44)	0.076
Delta WBC (cumm)	1.6 (0.2–3.7)	1.3 (-0.1-3.0)	1.8 (0.4–4.6)	0.225
Delta N/L ratio	1.4 (0.4–3.8)	1.2 (0-2.7)	3.1 (0.7–4.4)	0.047
Delta CRP (mg/dl)	1.75 (0.77–3.01)	1.16 (0.51-2.00)	2.68 (1.36–4.1)	0.009
Total time (min)	20.5 (18-27.3)	20 (18–28)	21 (17–27)	0.869
PEG procedure time (min)	9 (6–10)	9 (6.5–11)	8 (6-9.5)	0.068
Fever (%)	3 (6%)	0	3 (12%)	0.235
Total peristomal wound infection (%)	10 (20%)	6 (24%)	4 (16%)	0.725
Total pneumonia (%)	5 (10%)	1 (4%)	4 (16%)	0.349
Total stomal leakage (%)	8 (16%)	5 (20%)	3 (12%)	0.702
All-cause infection (%)	13 (26%)	6 (24%)	7 (28%)	0.725
Admission days	6.5 (4–11)	7 (4-10.5)	6 (4–13)	0.770

### Factors associated with infection

**Fig. 3:**
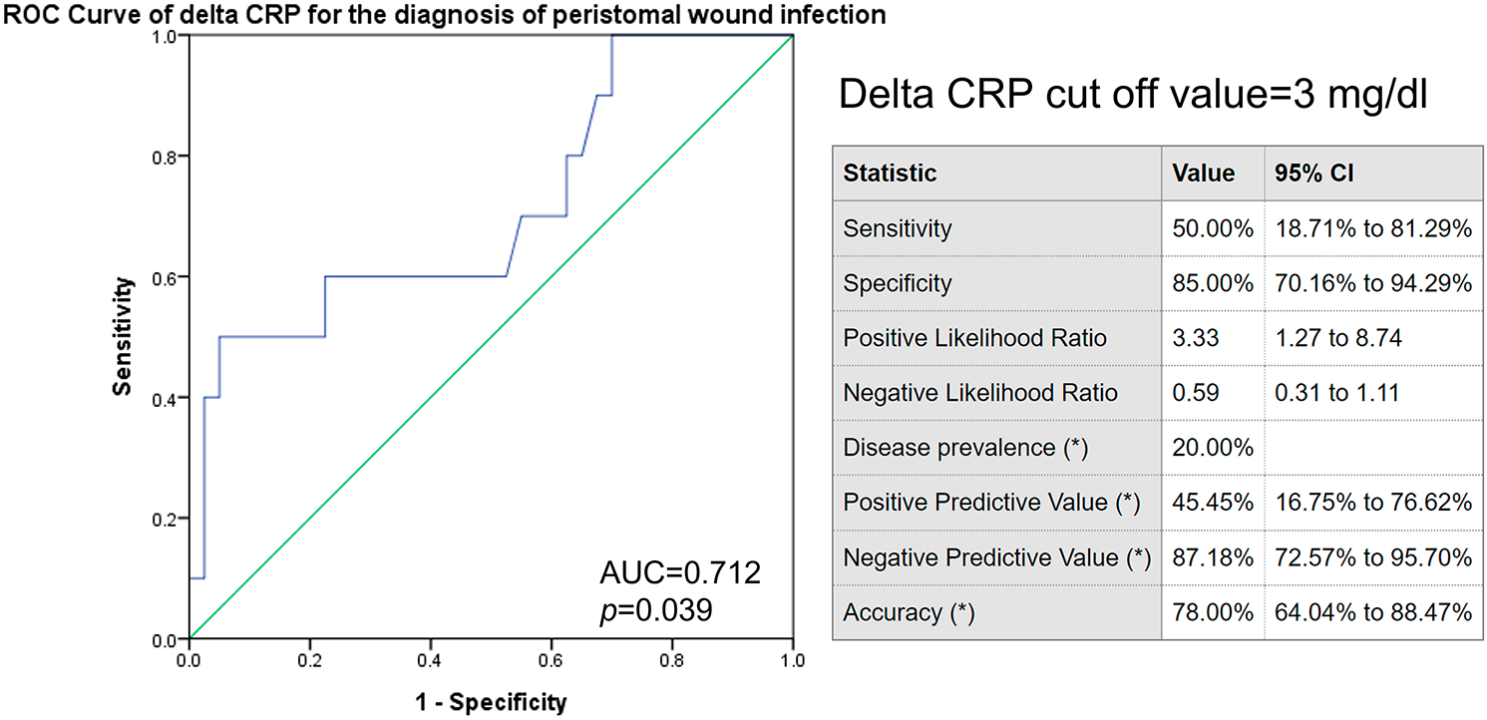
ROC curve of delta CRP for the diagnosis of peristomal wound infection

**Fig. 4:**
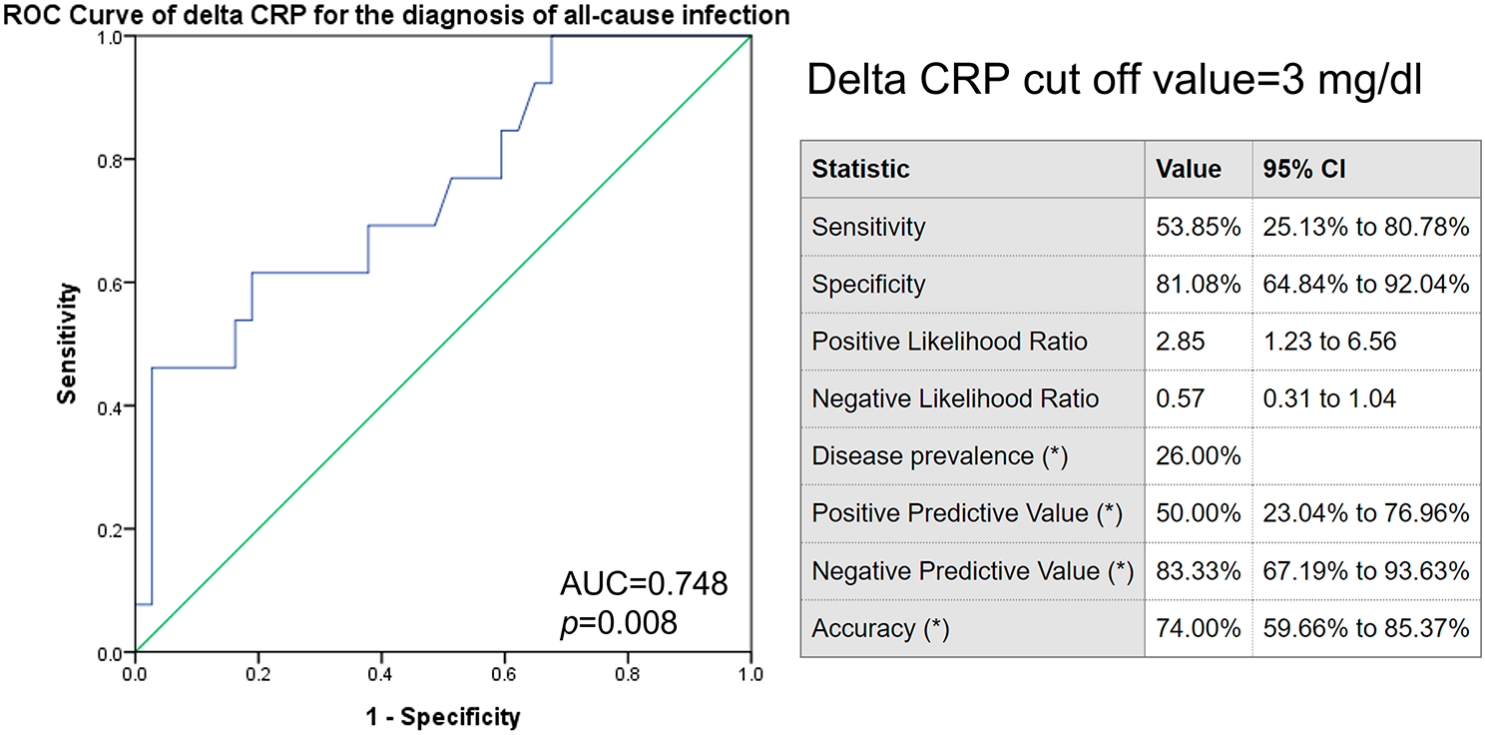
ROC curve of delta CRP for the diagnosis of all-cause infection

On univariable analysis of 50 patients undergoing PEG, Delta CRP was the only predictive factor of peristomal infection (Table 2). Delta CRP and hospitalization days were associated with higher all-cause infection rates in univariable analysis, but only Delta CRP independently predicted all-cause infection in multivariable analysis (Table 3). The ROC curve of Delta CRP for the diagnosis of peristomal wound infection showed an area under the ROC of 0.712 (p = 0.039) (Fig. 3). The best cut-off points of Delta CRP for the diagnosis of peristomal wound infection was 3 mg/dl, with a sensitivity of 50%, specificity of 85%, PPV of 45.45%, NPV of 87.18%, and accuracy of 78% (Fig. 3). Meanwhile, the ROC curve of Delta CRP for the diagnosis of all-cause infection showed an area under the ROC of 0.748 (p = 0.008) (Fig. 4). The best cut-off points of Delta CRP for the diagnosis of all-cause infection was 3 mg/dl, with a sensitivity of 53.85%, specificity of 81.08%, PPV of 50%, NPV of 83.33%, and accuracy of 74% (Fig. 4).

**Table 2:** The univariate and multivariate analysis of peristomal infection

Peristomal infection	Univariate analysis		Multivariate analysis	
Variable	Hazard ratio (95% CI)	*p*	Hazard ratio (95% CI)	*P*
Age (y/o) > 65/≦65	1.227 (0.300-5.028)	0.776		
Gender M/F	1.909 (0.431–8.463)	0.395		
BMI	0.949 (0.794–1.133)	0.560		
Head and neck cancer	1.750 (0.280-10.702)	0.545		
CVA	1.385 (0.332–5.773)	0.655		
HTN	0.739 (0.184–2.965)	0.670		
DM	0.231 (0.026–2.020)	0.185		
Dementia	1.385 (0.332–5.773)	0.655		
ALS	0.519 (0.196–2.799)	0.466		
Betadine	1.833 (0.448–7.511)	0.400		
Albumin (g/dl)	0.973 (0.308–3.075)	0.962		
Baseline glucose (mg/dl)	0.986 (0.959–1.014)	0.340		
Baseline WBC (cumm)	0.750 (0.493–1.140)	0.178		
Baseline CRP (mg/dl)	0.957 (0.530–1.731)	0.886		
Delta WBC (cumm)	1.153 (0.944–1.408)	0.162		
Delta CRP (mg/dl)	1.731 (1.122–2.669)	0.013	1.731 (1.122–2.669)	0.013
Delta N/L ratio	1.035 (0.881–1.216)	0.678		
Total time (min)	0.997 (0.892–1.116)	0.964		
PEG procedure time (min)	1.095 (0.900-1.331)	0.366		
Hospitalization days	1.016 (0.937–1.102)	0.699		

**Table 3:** The univariate and multivariate analysis of all-cause infection

All-cause infection	Univariate analysis		Multivariate analysis	
Variable	Hazard ratio (95% CI)	*p*	Hazard ratio (95% CI)	*P*
Age (y/o) > 65/≦65	2.132 (0.557–8.162)	0.269		
Gender M/F	1.219 (0.335–4.441)	0.764		
BMI	0.937 (0.796–1.104)	0.437		
Head and neck cancer	1.164 (0.197–6.881)	0.867		
CVA	1.302 (0.351–4.837)	0.692		
HTN	0.584 (0.164–2.087)	0.408		
DM	0.379 (0.072–1.985)	0.251		
Dementia	0.494 (0.135–1.808)	0.287		
ALS	0.336 (0.064–1.749)	0.195		
Betadine	0.905 (0.255–3.211)	0.877		
Albumin (g/dl)	1.022 (0.356–2.931)	0.967		
Baseline glucose (mg/dl)	0.979 (0.952–1.008)	0.151		
Baseline WBC (cumm)	0.736 (0.501–1.080)	0.117		
Baseline CRP (mg/dl)	1.008 (0.597-1.700)	0.977		
Delta WBC (cumm)	1.205 (0.991–1.465)	0.061		
Delta CRP (mg/dl)	1.756 (1.166–2.645)	0.007	1.709 (1.122–2.604)	0.013
Delta N/L ratio	1.109 (0.953–1.291)	0.181		
Total time (min)	0.974 (0.876–1.083)	0.621		
PEG procedure time (min)	1.009 (0.834–1.222)	0.923		
Hospitalization days	1.115 (1.004–1.239)	0.041	1.101 (0.989–1.225)	0.078

### Analysis of adverse event

One patient expired 6 days after PEG implantation due to pneumonia. Tracing back record, the patient experienced sudden dyspnea and high fever after feeding. Milk-like material was noted after airway suction and aspiration was impressed. Thirteen patients experienced infection episodes after PEG implantation within 2 weeks. Eight had peristomal wound infection, three of them had pneumonia, and two of them had both (Supplementary Table 1). Of the 13 patients, the results of mouth culture before PEG implantation represented dominant microbes of infection in nine patients (69.2%). Common pathogens included *Pseudomonas aeruginosa, Escherichia coli*, and *Klebsiella pneumoniae.* Two patients expired after PEG implantation within 1 month due to pneumonia. One was in the Betadine group, and the other was in the control group.

## Discussion

This is the first study to assess the efficacy of Betadine coating for preventing PEG peristomal infection. Our finding demonstrated that Betadine coating could not prevent peristomal infection or pneumonia. However, it could reduce serum level of CRP elevation after PEG. The microbes of mouth culture before PEG were highly correlated to microbes following infection, including pneumonia and peristomal wound infection. The Delta CRP at 24 h was the most important predictor of peristomal wound infection and all-cause infection within 14 days after PEG implantation.

Several studies discussed reducing the contact between the PEG tube and the organisms in the oral cavity. Maetani et al. [10] found that overtube implantation could reduce peristomal wound infection after PEG implantation. Horiuchi et al. [17] found that intranasal application of mupirocin, arbekacin inhalation, and oral sulfamethoxazole/trimethoprim for methicillin-resistant Staphylococcus aureus (MRSA) decolonization in the oral cavity could reduce peristomal wound infection.

However, in our study, we found Betadine coating on PEG tubes did not reduce peristomal wound infection. Previous studies have reported that Betadine irrigation before dental extraction or gingivectomy could reduce 30–40% of bacteremia [14, 15], but there is still a 20–30% prevalence of bacteremia in the Betadine irrigation group. It indicated that Betadine might be diminished by colonization but can not be eradicated.

Although Betadine coating could not reduce peristomal wound infection or pneumonia, we did find a lower elevation of serum CRP level and N/L ratio after PEG implantation in the Betadine group. According to previous studies, povidone-iodine has not only an antibacterial effect but also an anti-inflammatory effect. It can suppress human inflammatory effector cells and mediators of inflammation such as TNF-α and β-galactosidase [18, 19]. Meanwhile, CRP is synthesized in the inflammatory response to interleukin-6 and regulated by TNF-α [20]. Changes in serum CRP levels are not only a diagnostic tool for sepsis and infection [21, 22] but also an indicator of inflammation evolution. According to our findings, the Delta CRP before and after PEG implantation could independently predict following peristomal wound infection and all-cause infection, with the best cut-off value of Delta CRP 3 mg/dl. It is of note that Betadine coating on PEG tube could reduce the elevation of serum CRP level after PEG implantation, but not peristomal wound infection rate. It is believed that Betadine coating on PEG tube might decrease microbes’ colonization and diminish inflammatory response, but not enough to reduce infection. A previous article suggested povidone-iodine ointment over the stomal wound after PEG implantation [23], the anti-inflammatory effect might also be effective locally, but only limited 379 at skin site. However, Delta CRP 3 mg/dl had a good NPV for peristomal wound infection and all-cause infection as a cut-off point. Also, the organisms of mouth culture were identical to those following wound and sputum culture. This indicates that oral microbes represent most of the peristomal infection and pneumonia after PEG. Therefore, oral disinfection can not be overemphasized before performing PEG. Pre-emptive antibiotics according to mouth culture might be considered in patients with delta CRP > 3 mg/dl.

There are several limitations in our study. First, it is a single center randomized controlled trial with small sample size. Second, there was no positive finding on the primary outcome, which might be owing to small sample size and inappropriate estimation of power. However, the post hoc analysis demonstrated that serum CRP change after PEG implantation could predict subsequent infection, which might be beneficial for clinical practice.

In conclusion, a Betadine coating gastrostomy tube can reduce CRP elevation after PEG implantation but can not reduce peristomal infection. The change of serum CRP level after PEG can predict peristomal wound infection and pneumonia. The microbes of mouth culture are highly correlated with the pathogen of subsequential pneumonia and wound infection. The efficacy of povidone-iodine coating gastrostomy tube should be re-evaluated under an appropriately powered study in the future. Whether pre-emptive antibiotics are needed to abort potential infection in patients with elevation of CRP after PEG is deserved further study.

## Electronic supplementary material

Below is the link to the electronic supplementary material.

Supplementary table 1: Bacterial culture results in infected patients

## Data Availability

The datasets used during the current study are available from the corresponding author upon reasonable request.
