# Supplementary material for: Effectiveness of betadine-coating gastrostomy tube to reduce peristomal infection after percutaneous endoscopic gastrostomy: a randomized controlled trial
Source: BMC Gastroenterol. 2023 May 15;23:155. doi: 10.1186/s12876-023-02702-w (PMC10186665; doi:10.1186/s12876-023-02702-w)
Supplement: Supplementary file 1 — Supplementary table 1: Bacterial culture results in infected patients [file 12876_2023_2702_MOESM1_ESM.docx]

Supplementary table 1 Bacterial culture results in infected patients

|  | **Mouth culture** | **Wound culture (with stomal infection)** | **Sputum culture (with pneumonia)** |
| --- | --- | --- | --- |
| Case 1 (control) | Pseudomonas aeruginosa | Pseudomonas aeruginosa |  |
| Case 2 (control) | Pseudomonas aeruginosa, Escherichia coli | Escherichia coli、GPB |  |
| Case 3 (betadine) | Pseudomonas aeruginosa | Pseudomonas aeruginosa |  |
| Case 4 (betadine) | No bacterial growth | No bacterial growth | Staphylococcus aureus |
| Case 5 (betadine) | GNB | No bacterial growth |  |
| Case 6 (betadine) | Enterobacter cloacae complex/Acinetobactor baumannii complex | Acinetobactor baumannii complex |  |
| Case 7 (betadine) | Klebsiella pneumoniae(CRE) | No bacterial growth |  |
| Case 8 (control) | GNB |  | GNB |
| Case 9 (control) | Pseudomonas aeruginosa |  | Pseudomonas aeruginosa |
| Case 10 (betadine) | Corynebacterium striatum | No bacterial growth |  |
| Case 11 (control) | Klebsiella pneumoniae/Staphylococcus aureus |  | Klebsiella pneumoniae/Staphylococcus aureus |
| Case 12 (control) | Escherichia coli | GNB | Escherichia coli |
| Case 13 (control) | Klebsiella pneumoniae | Klebsiella pneumoniae |  |
